# Medical and Surgical Treatment in Pediatric Orbital Myositis Associated with Coxsackie Virus

**DOI:** 10.1155/2015/917275

**Published:** 2015-10-15

**Authors:** Pedro Gil, João Gil, Catarina Paiva, Guilherme Castela, Rui Castela

**Affiliations:** Department of Ophthalmology, Centro Hospitalar Universitário de Coimbra, Praceta Professor Mota Pinto, 3000-075 Coimbra, Portugal

## Abstract

*Purpose.* To report a case of orbital myositis associated with Coxsackie virus and its medical and surgical approach. *Methods.* Complete ophthalmological examination and imaging and analytical investigation were performed. *Results*. A 6-year-old male presented with subacute painless binocular horizontal diplopia. Examination revealed bilateral best-corrected visual acuity (BCVA) of 20/20 and right eye 45-prism-dioptre (PD) esotropia in near and distance fixations, with no motility restrictions. Serologic screening was positive for Coxsackie virus acute infection and computerized tomography (CT) suggested right eye medial rectus orbital myositis. An oral corticosteroid 1.0 mg/kg/day regimen was started. A new CT after two months showed symmetrical lesions in both medial rectus muscles. Corticosteroids were increased to 1.5 mg/kg/day. After imagiological resolution on the 4th month, alternating 45 PD esotropia persisted. Bilateral 7 mm medial rectus recession was performed after 1 year without spontaneous recovery. At 1-year follow-up, the patient is orthophoric with 200′′ stereopsis and bilateral 20/20 BCVA. *Conclusions*. To our knowledge, this is the first reported case of orbital myositis associated with Coxsackie virus. This is also the first reported case of isolated strabismus surgery after orbital myositis in pediatric age, highlighting the favourable aesthetic and functional outcomes even in cases of late ocular motility disorders.

## 1. Introduction

Idiopathic orbital inflammatory syndrome (IOIS), formerly termed orbital pseudotumor, is a benign idiopathic inflammatory disease that can affect any structure of the orbit. Previous authors have reported that it accounts for 5.0–6.3% of orbital disorders, being the third most common after thyroid associated orbitopathy and lymphoproliferative disease [1].

According to the current most consensual classification, orbital myositis represents a subgroup within the IOIS, as an inflammatory process that primarily involves the extraocular muscles [2]. The relative incidence of this subtype to the IOIS group is variable in different case series, ranging from 29% to 50% [3, 4].

Some authors have identified two variants, the first and most common being idiopathic and the second considered to be specific [2]. The mechanisms for disease are not fully understood, although a significant role for the immune system response to some bacterial or viral antigens and endotoxins has been proposed [5]. In other patients, orbital myositis can be related to a heterogeneous group of specific diseases, including bacterial or viral infections and systemic immune-mediated diseases. Therefore, the classification of myositis as idiopathic is a diagnosis of exclusion and can only be assumed after a complete systemic investigation.

We report an atypical presentation and successful surgical outcome for this rare disease, describing for the first time an association with Coxsackie virus infection.

## 2. Case Presentation

**Figure 1:**
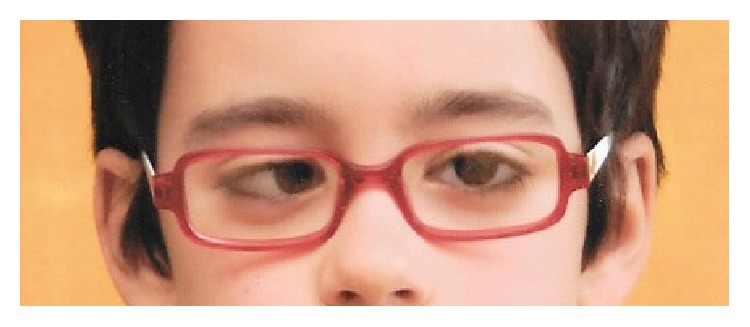
Right eye esotropia in primary gaze position at presentation.

A 6-year-old male patient reported to the Emergency Room of our tertiary care paediatric center complaining of binocular horizontal diplopia, with a subacute onset lasting for one week. Complete ophthalmological examination revealed a best-corrected visual acuity (BCVA) of 20/20 on both eyes and significant right eye (RE) esotropia, measuring 45 prism dioptres (PD) both in near and in distance fixation (Figure 1). No apparent restriction was present in terms of ocular motility. The patient denied any orbital pain and there was no conjunctival injection, proptosis, ptosis, or any other abnormality in anterior segment examination of both eyes. Fundoscopic examination was unremarkable.

**Figure 2:**
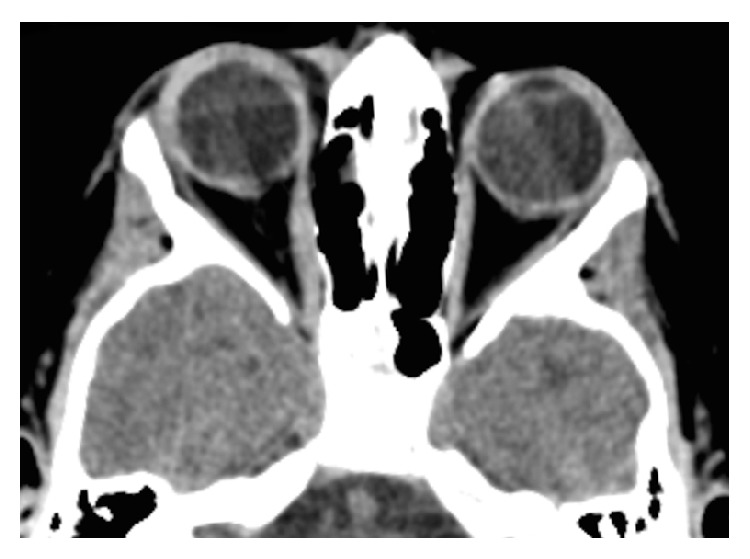
Brain computerized tomography at presentation showing thickening of the right eye medial rectus muscle, associated with increased spontaneous density.

Complete blood count was normal, PCR was negative, and the erythrocyte sedimentation rate was slightly increased at a value of 18 mm/hour. Complete serologic screening was performed for toxoplasmosis, cytomegalovirus, herpes simplex viruses 1 and 2, Epstein-Barr virus, and Coxsackie virus. All results were negative for acute infection, except for Coxsackie virus which showed positive IgG 559.00 U/mL and IgM 68.90 U/mL counting, compatible with recent infection. The analysis was repeated 2 weeks later, when it showed a consistent positive result for acute infection: IgG 1488.00 U/mL and IgM 95.60 U/mL. Thyroid hormone tests (TSH, free T3, and free T4) were normal. Brain and orbital computerized tomography (CT) showed discrete thickening of the RE medial rectus muscle, associated with increased spontaneous density and diffuse enhancement after intravenous contrast administration (Figure 2). No other abnormality was identified and the remaining extraocular muscles were normal.

The diagnosis of orbital myositis associated with Coxsackie virus was suspected and the patient was initiated on an oral corticosteroid treatment with a dosage of 1.0 mg/kg/day. He was also started on a 3-hour daily regimen of patching of the left eye (LE).

**Figure 3:**
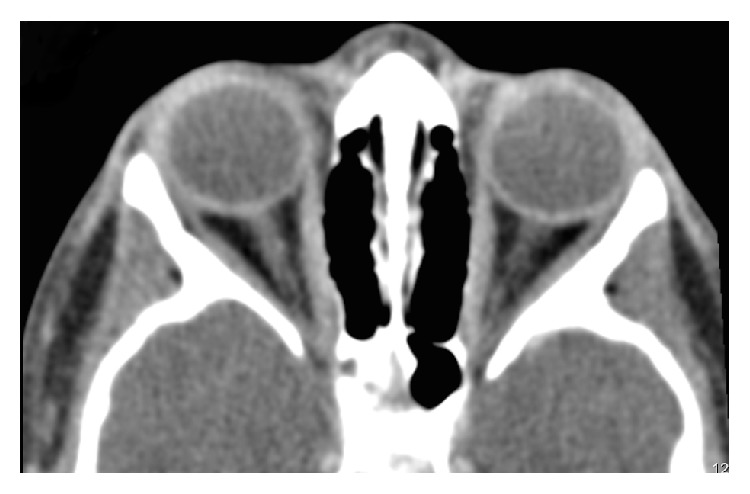
Brain computerized tomography at 2 months showing bilateral thickening of the medial rectus muscles, associated with increased spontaneous density.

The patient was reevaluated 2 and 4 weeks after the onset of disease, with no significant clinical improvement. Two months later, while the patient was still under the initial oral corticosteroid regimen, complete ophthalmological examination was similar to the initial observation, except for the BCVA of the RE, which had decreased to 20/25. A new brain and orbital CT was performed. It showed overlapping characteristics comparing to the previous exam, but this time symmetrical: both right and left medial rectus were thickened comparing to the other extraocular muscles, associated with increased spontaneous density and diffuse enhancement after intravenous contrast administration (Figure 3). Comparing to the first performed CT, there had been no significant reduction of the RE medial rectus' thickness.

Given the absence of a favourable response to the initial corticosteroid regimen, the daily dosage was increased to 1.5 mg/kg.

**Figure 4:**
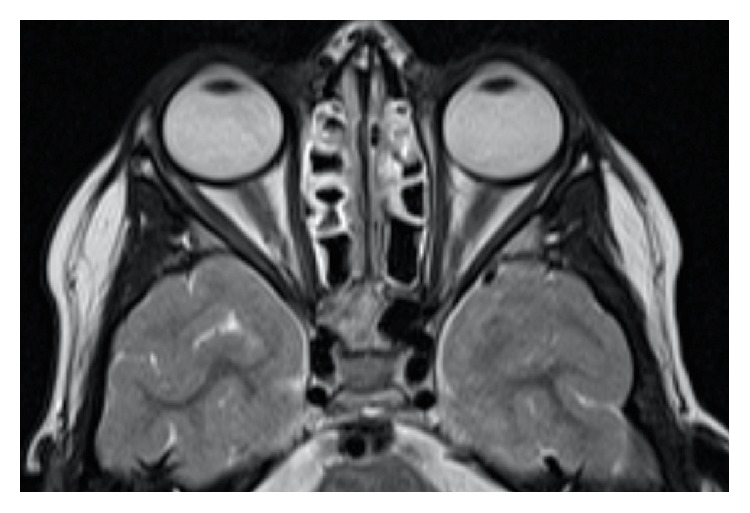
Brain magnetic resonance at 4 months showing a significant reduction of the right and left eyes medial rectus' thickness.

Four months after the onset, BCVA improved to 20/20 on both eyes, while the remaining ophthalmological examination still revealed 45 PD esotropia of the right eye, both in near and in distance fixation, with no stereopsis on the Lang II test. The patient underwent brain and orbital magnetic resonance (MR) that showed a significant reduction of the right and left eyes medial rectus' thickness, with no significant differences before and after contrast administration compared with the other extraocular muscles (Figure 4). No other lesions were present.

The patient still complained of binocular horizontal diplopia. Given the symmetrical BCVAs, we opted for an alternate occlusion strategy, with good compliance.

Eight months after the onset, new MR showed no recurrence of inflammatory lesions. The patient denied binocular diplopia, and suppression of the RE was evident with Worth four-dot testing. Ophthalmological examination revealed alternating esotropia, measuring 45 PD both in near and in distant fixation.

**Figure 5:**
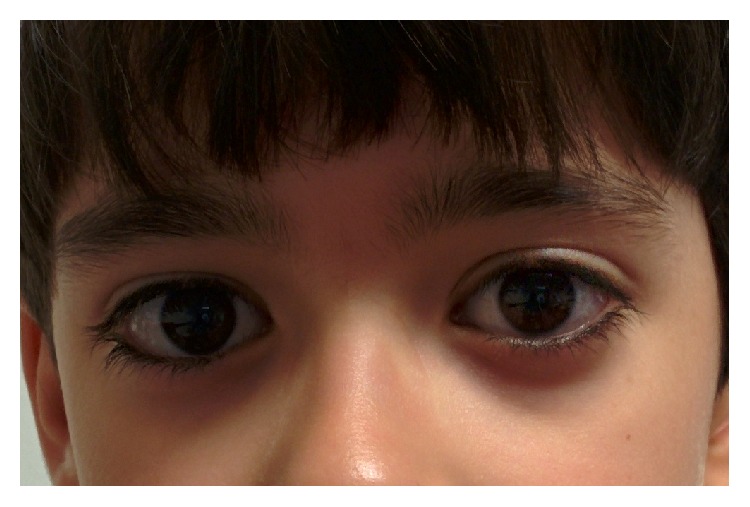
Primary gaze position orthophoria at 6 months postoperative follow-up.

Given the absence of spontaneous recovery of the ocular motility disorder 1 year after the onset of disease, we opted for surgical treatment: bilateral 7 mm medial rectus recession. The 6-week postoperative visit showed an orthophoric patient in all positions of gaze, with no complaints of diplopia and with a BCVA of 20/20 on both eyes (Figure 5). Stereopsis measurement was 200′′ on Lang II test.

With 1 year of postoperative follow-up, making a total of 2 years after the disease onset, the patient is still orthophoric, with a BCVA of 20/20 on both eyes and with no evidence of recurrence of disease both clinically and in imaging exams.

## 3. Discussion

Since the first report of orbital myositis associated with systemic diseases, multiple case reports have been published where the causal relationship between orbital myositis and a specific inflammatory or infectious disease is circumstantial. This is also true in our case. It is difficult to ascertain a definite causal relationship because the exact pathogenic mechanisms are still widely unknown. Therefore, the association is based on the coexistence of a clinical diagnosis of orbital myositis and a systemic condition that can predispose to an inflammatory process affecting the extraocular muscles.

Not only is orbital myositis considered a rare disease* per se*, but also the clinical case presented is atypical in many ways. In terms of epidemiology, children are not frequently affected, although when they are, bilateral disease is common, as it was in our patient [6]. Clinical presentation was unspecific, and the absence of orbital pain in eye movements, conjunctival injection, ptosis, or any other acute sign was a confounding factor for the correct diagnosis. The fact that diplopia was the only presenting sign obligates us to include orbital myositis in the differential diagnosis of acute-onset diplopia.

As for the complementary examination, the findings on CT imaging were typical, and alongside MR it proved useful not only for diagnosis but also for monitoring treatment response.

Previous reports have linked Coxsackie virus infection to myositis [7]. To our knowledge, this is the first reported case of orbital myositis associated with Coxsackie virus. It is still unclear whether viral or bacterial-associated orbital myositis differs from the idiopathic type in terms of clinical outcome or treatment. However, it is important to recognize all possible pathogens, not only to guide systemic investigation but also to expand our knowledge in terms of possible etiopathogenic associations.

According to current consensus, the patient was started on an oral corticosteroid daily regimen of 1.0 mg/kg. This dosage proved insufficient and given the evidence of bilateral involvement we increased the dosage to 1.5 mg/kg/day, which is the maximum proposed by most authors [2, 8]. It is not clear whether this occurred due to some potential for local dissemination of the inflammatory process or whether the inflammatory response in the LE medial rectus was deferred by any defensive mechanism. Asymmetric disease has been reported in the idiopathic type [9], but the reasons behind asymmetric and delayed onset disease in one eye assuming a systemic infectious aetiology are unclear. Like previous authors, we suggest that an aggressive and early disease therapy might be beneficial [2]. This could prevent local dissemination to other extraocular muscles and orbital structures, thus maybe reducing duration of disease and late stage sequelae.

This case also highlights the importance of an integrated approach to treatment. Reducing inflammation is primordial, but accounting for all the comorbidities is also important. In our case, the resulting strabismus was probably due to cicatricial fibrotic contraction of medial rectus muscles, leading to esotropia. Therefore, prevention of strabismic amblyopia is of paramount importance. Most authors reported prompt clinical improvement and remission within days to weeks after treatment in most patients [8]. Our case is atypical in the way that inflammation was adequately controlled with the recommended corticosteroid regimen, but the late stage sequelae of alternating strabismus posed a challenge. Given the rarity of this entity in children in the amblyopia age interval and the favourable outcome in most patients, there is scarce reference in the published literature regarding treatment in cases of ocular motility disorders secondary to orbital myositis in paediatric age. Bessant and Lee published a case series of 5 consecutive patients who had suffered one or more episodes of orbital myositis resulting in a persistent ocular motility defect associated with significant diplopia [10]. Only one of these patients was in paediatric age (11 years). Botulinum toxin A (BTXA) was injected in all patients, after 3 months of evidence of absence of improvement in the ocular motility status. According to the authors, BTXA not only is aimed at improving symptoms of diplopia, but can also be used to determine whether a patient has the potential to achieve binocular single vision after a corrective strabismus surgery. Surgery was indeed performed in 2 of the 5 patients, including the one in paediatric age.

To our knowledge, this is the first reported case of isolated strabismus surgery after orbital myositis in paediatric age. We still consider as a valid approach the use of BTXA as a conservative first-line therapy prior to corrective surgery. However, it is noteworthy to see that in our case medial rectus recession after 1 year of onset provided not only an excellent aesthetic outcome, but also a surprisingly good functional outcome, as the patient recovered a significant degree of stereopsis after binocular alignment. Given the lack of published reports, there is no consensus regarding the outcomes after BTXA or strabismus surgery and whether they are dependent on the duration of disease, degree and type of deviation, number and type of affected extraocular muscles, laterality, age, and other factors. According to these clinical features, for which published case reports can give a significant contribution, future guidelines might clarify the role for BTXA or surgery in these patients (with special consideration to those in the amblyopia age interval) and the adequate timing for each intervention.
